# Embodied cognition perspectives within early executive function development

**DOI:** 10.3389/fcogn.2025.1361748

**Published:** 2025-02-18

**Authors:** Z. Reagan Pearce, Stephanie E. Miller

**Affiliations:** Department of Psychology, University of Mississippi, Oxford, MS, United States

**Keywords:** childhood, executive function, representation, embodied cognition, information-processing

## Abstract

The development of executive function (EF) has become a central focus in early cognitive development research. While movement is frequently used to measure EF in young children and may significantly contribute to its development, many leading EF theories do not fully explore the role of movement. This review investigates the critical role of movement in the development of EF during early childhood through the framework of embodied cognition, particularly drawing on the central themes outlined by Lawrence Shapiro. By applying Shapiro's themes, this narrative review examines whether and how these embodied cognition concepts are integrated into leading theories of EF development. The analysis identifies key gaps where current theories could benefit from a deeper incorporation of embodied cognition. This work aims to support future research that emphasizes the importance of movement in fostering EF during early childhood.

## 1 Introduction

Young children rely heavily on motor skills and bodily interaction with the environment to communicate, learn, and explore (Piaget, 1959; Thelen and Smith, 1994; Hohmann et al., 1995; Pellegrini and Smith, 1998). Observing children's bodily actions and goal-directed behaviors forms a cornerstone of studying cognition in the first few years of life as motor skill development and cognition are intrinsically intertwined. Despite the significance of motor skill development in understanding the development and emergence of cognitive processes during preschool years, relatively few theoretical perspectives focus on the role of motor actions and bodily capabilities in the development of controlled cognition, specifically termed executive function (EF). EF, encompassing skills such as working memory, inhibitory control, and cognitive flexibility, undergoes rapid development during the preschool years (3–5 years; Zelazo and Carlson, 2012), a period still heavily influenced by movement and the transition from motor-based thought to symbolic thought. Thus, considering the specific relationships between EF, motor skill development, and bodily interaction with the environment is crucial (Lux et al., 2021). This paper addresses the research question: How do radical embodied cognition theories inform our understanding of executive function (EF) development in early childhood, and how do they complement or challenge existing EF frameworks?

While perspectives emphasizing the role of bodily action and motor skill competence within cognition often fall under the research program known as embodied cognition (Shapiro and Stolz, 2019; Varela et al., 2016), there is limited integration of these theories within developmental EF models. Embodied cognition research often suggests that EF and corresponding goal-directed behaviors are grounded in the ability to plan and control motor actions (Gottwald et al., 2016; Ridler et al., 2006). However, the field of embodied cognition is broad with somewhat vague and varied definitions (Da Rold, 2018; Shapiro and Spaulding, 2021), which complicates its application to EF theory. This review focuses specifically on Lawrence Shapiro's work, which provides three concrete concepts for defining EF (Shapiro, 2011, 2012; Shapiro and Stolz, 2019). While Shapiro's framework is not the only approach within embodied cognition and may not be the predominant theory, it offers a manageable basis for comparison with EF theories.

The aim of this review is to critically evaluate how embodied cognition, particularly Shapiro's work, integrates into existing developmental theories of EF. We assess how these perspectives complement or challenge traditional EF frameworks and summarize the implications of these differences for understanding EF development in early childhood. By providing a detailed comparison of the key concepts in both EF and embodied cognition, we offer a critical analysis of how motor skills and body-environment interactions contribute to the development of EF. This review also distinguishes key terms—movement, action, motor skill development, and body—to provide a more nuanced foundation for the discussion (Lux et al., 2021). Through this lens, the paper explores the interplay between movement and EF, emphasizing how their mutual influence shapes cognitive growth in preschool children. By integrating radical embodied cognition with traditional EF theories, we offer new insights into how physical movements and sensory experiences fundamentally shape EF development, highlighting the importance of including embodied perspectives in early EF research.

While cognitive development encompasses a range of mental processes and skills, this review specifically focuses on how body-environment interactions and motor skill development contribute to the development of executive functions (EF). It offers a unique perspective on the interplay between movement and EF, highlighting their mutual influence on cognitive growth. We concentrate on early childhood, particularly children aged 3 to 5 years, while also referencing broader EF theories that extend into later childhood and adulthood for a comprehensive view. The review explores how mental representation, embodied cognition, and information-processing perspectives enhance our understanding of EF in preschool children. By examining various theories—including neurocognitive, attentional, representational, and dynamic systems theories—this review contextualizes EF development during the preschool years. This approach introduces a novel perspective by integrating radical embodied cognition with traditional EF theories, offering new insights into how physical movements and sensory experiences fundamentally shape EF development and highlighting the importance of emphasizing movement in early EF research.

## 2 Embodied cognition

Embodied cognition is often viewed as a challenger to traditional information-processing views of cognition. The latter compares the brain to a computer or calculator, translating sensory input into a code that the brain manipulates for behavioral output (Lachman et al., 2015; Shapiro, 2007). This manipulation, termed mental representation, forms the basis of cognition. According to this approach, the brain internally collects, stores, and modifies environmental information over time, with some processes taking longer than others (Lachman et al., 2015). The development of cognitive control and problem-solving processes is thought to occur internally with maturation of the brain, as suggested by many theories of EF development (Stuss and Benson, 1984; Diamond and Goldman-Rakic, 1985; Miyake et al., 2000; Moriguchi and Hiraki, 2013; Perone et al., 2018; Zelazo, 2020).

Recent research, however, has broadened the examination of cognition to include mechanisms outside of purely mental processes, suggesting a more significant role for the body and environment than traditional information-processing approaches suggest. While there is general acceptance of the influence of the body and environment on cognition, research diverges on how these factors interact to contribute to or replace mental processing (Wilson, 2002; Shapiro, 2011; Marshall, 2016). These divergent approaches replace the need for mental representation, representing extreme or radical views of embodied cognition (Wilson and Golonka, 2013; Chemero, 2011). Instead of viewing the brain as a computer that interprets and transmits codes to produce thought and action (Pellegrino and Goldman, 1987), embodied perspectives consider the brain as one component in a dynamic cognitive system.

An early illustration of radical embodied cognition's divergence from traditional information-processing models can be seen in Gibson's theory of perception (Gibson, 1979). Gibson proposed an alternative to the dominant explanation of visual perception by incorporating the mind, body, and their interactions with the environment. He hypothesized that perception is direct, meaning that enough environmental information is provided to obviate the need for computation within the brain. Perception, according to Gibson, is intended to guide action, enabling individuals to act in their environment. This direct perception relies on affordances, directly perceivable environmental opportunities, highlighting the importance of embodiment. Gibson's theory laid part of the foundation for radical embodied cognition by recognizing the role of action and environment in cognition.

Building on Gibson's foundational work, recent theorists have challenged the brain-as-computer hypothesis and expanded the scope of embodied cognition. Shapiro and Spaulding (2021) characterized embodied cognition as “a research program with no clear defining features other than the consensus that computational cognitive science has failed to appreciate the body's significance in cognitive processing…” (Shapiro and Spaulding, 2021). Although this definition does not clearly define components of embodied cognition, much of Shapiro's work (2011; 2012; 2019) has focused on identifying the central questions of embodied cognition (Martiny, 2011). These questions derive from three concepts (Table 1). Shapiro's first concept, **Conceptualization**, refers to the idea that concepts are formed through our interactions with the world, though it does not specify the neural mechanisms underlying this process. The next concept, **Replacement**, posits that mental concepts can be represented or simulated through bodily actions, suggesting that cognition can be achieved through embodied experiences. Finally, **Constitution** suggests that cognitive processes are embedded within the dynamic interactions between the body, environment, and neural systems.

**Table 1:** Approaches to answering questions related to embodied cognition (Shapiro, 2011).

	**Extended information-processing**	**Radical embodied cognition**
Relationship between body and concepts	*Development and changes of an organism's body (i.e., brain and sensory organs) influence the type of interaction that organism will have with the environment, therefore, acknowledging that the concepts an organism can acquire (and process) are dependent on changes to both body and environment (Lally and Valentine-French, 2019)*.	***Conceptualization**: An organism's body directly influences whether concepts can be acquired. The way an organism understands the environment depends on its body (Shapiro, 2011)*.
Need for representations	Human cognition is a stage-like system that processes environmental stimuli to execute behavior. The process begins with input from the environment via senses. Relative information is then processed and encoded as representations that can be stored and manipulated later on. The role of body and action in information-processing models may help explain how organisms form and maintain mental representations. The interaction between the body and environment is influential to mental processing.	***Replacement**: There is a need to completely replace computational concepts like symbol, representation, and inference with bodily-informed cognitive systems. An organism's body and actions do enough of the work required to achieve higher-level goals, replacing the need for complex mental representations (Wilson and Golonka, 2013; Shapiro and Spaulding, 2021)*.
Mechanisms Involved in Cognition	Cognition consists of higher-level functions of the brain. The body and environment serve as secondary resources for acquiring input for internal structures to process. The roles of the body and interactions with the environment can guide internal structures (Clark, 1999).	***Constitution**: The brain is inherently linked with the environment, such that the input and output systems are integrated, as a dynamic system, rather than as separated elements. The body and environment are a part of the cognitive system (Chiel and Beer, 1997; Shapiro and Spaulding, 2021)*.

*Italic font indicates that the component is met;* Upright font indicates that the component is not met.

Thus, radical embodied cognition addresses three central questions: the relationship between the body and concepts as mental objects, the need for mental representation to reconstruct stimuli from the environment, and what mechanisms should be considered part of the cognitive system (Shapiro, 2011). These questions serve as a starting point for establishing how embodied perspectives fit into existing theories of cognitive development.

### 2.1 Extending information-processing through embodiment

Many current information-processing approaches address aspects of the three questions posed by Shapiro's radical embodied cognition (see Table 1; Shapiro, 2011). However, these approaches tend to prioritize internal processing states (i.e., mental representations) even while engaging with questions similar to those raised by radical embodied cognition. For instance, Wilson (1994) proposed “wide computationalism,” which suggests that cognition is a byproduct of an information-processing mechanism that extends beyond the brain. According to this view, the brain can offload certain mental tasks onto the environment—such as writing a grocery list on paper instead of memorizing it. Critics, however, argue that while such approaches acknowledge the body's role, they do not constitute true embodied cognition. Instead, they represent forms of “situated,” “extended,” or “embedded” cognition. For example, wide computationalism resembles extended cognition, where cognitive processes are externalized to the environment, aiding in problem-solving and task execution. Situated cognition, on the other hand, posits that while cognitive processes occur within the brain (e.g., formation and storage of mental representations), the body's engagement with the environment continuously feeds information back into these processes, influencing behavior and action (Wilson, 2002). Meanwhile, embedded cognition suggests that cognitive processes within the brain are influenced by external factors such as culture, environment, and other organisms (Clark, 2008; Hutchins, 1995; Kirsh, 1995, 2010; Wilson, 2002).

Despite these differing perspectives, the integration of embodied cognition into information-processing theories has led many researchers to explore how movement and environmental factors contribute to the building, maintaining, and updating of mental states. This “softer” interpretation of embodied cognition underscores the crucial role of the body and environment in cognition, challenging traditional information-processing models that may have overlooked their significance (Shapiro, 2011; Clark, 1999).

While radical embodied cognition opposes the notion of mental representation, it currently does not offer a clear alternative role for the brain. Thus, while information-processing approaches cannot be entirely replaced by radical embodied cognition, there is potential to integrate radical views with extended information-processing theories to gain a deeper understanding of the interplay between the mind, body, and environment in cognition.

### 2.2 Embodied cognition in early development

One area where radical embodied cognition and extensions of information-processing can provide insight is in EF development, particularly during preschool when children are rapidly developing both motor and cognitive skills (Thelen et al., 2001; Smith and Gasser, 2005). Children at this age are transitioning from learning primarily through active engagement with the environment. Therefore, there has been an increase in work aimed at understanding how the body and movement within the environment can improve social, academic, and cognitive domains for children.

Although early theories of EF development, such as those by Piaget, acknowledged the importance of a child's actions and interactions with their environment, they may not fully encapsulate the principles of embodied cognition. Piaget's sensorimotor stage (0 to 2 years) highlights how infants gain knowledge by actively engaging with their surroundings, developing mental representations through these interactions (Piaget, 1959, 1971). However, as children transition to the preoperational stage, Piaget suggested a shift toward symbolic thinking, with less emphasis on physical interaction (Piaget, 1936, 1959).

In contrast, more recent research within the framework of embodied cognition emphasizes the continuous and dynamic interaction between motor development and cognitive processes across all stages of development. Hoch and Adolph (2019) provide a comprehensive review of motor development, illustrating how motor skills are integral to cognitive development from infancy through childhood. Their work, along with that of Lockman, Thelen, and Smith, underscores the perception-action perspective, which posits that EF development is deeply intertwined with the ability to perceive and act within one's environment (Lockman, 2000; Thelen and Smith, 1994). For example, Thelen's dynamic systems theory (1994) argues that motor development is not just a backdrop for cognitive development but a central component that influences and is influenced by cognitive processes. This theory suggests that the development of motor skills can provide the scaffolding for the emergence of more complex cognitive functions. Additionally, Vygotsky's cognitive development theory also acknowledged the importance of active engagement with the environment, emphasizing the sociohistorical context and the role of social interaction in cognitive development (Vygotsky, 1962, 1978). However, Vygotsky's theory still prioritized mental representations and symbolic thought.

Marshall (2016) advocates for an embodied perspective of human development through a systems approach, emphasizing that the mutually influential interactions between the agent and the context are what make development adaptive (Lerner and Schmid Callina, 2013; Brandtstädter, 1998). This perspective aligns with the concept of plasticity, which encourages systematic change within the interaction between the environment and the agent. By integrating these perspectives, we can better understand how the body and environment play a crucial role in the development of executive function (EF) during the preschool years.

### 2.3 Embodied cognition in early executive function development

The literature has increasingly examined embodied perspectives within the development of conscious control of thoughts, behaviors, and actions, recognizing the crucial role of the body and environment in EF development. Preschool age (3–5 years) has been of particular interest to early EF researchers, as it is a period of rapid development for both EF and motor skills, with implications for social competence, academic achievement, and later quality of life (Diamond, 2013; Lerner et al., 2017; Devine et al., 2019). This section will explore how embodied cognition provides a more comprehensive framework for understanding the interplay between motor and cognitive development, moving beyond the limitations of traditional information-processing models.

Embodied frameworks offer a promising avenue to enhance current explanations of EF skill emergence by considering the interaction between the mind, body, and environment in cognition. For example, Gottwald et al. (2016) demonstrate how movement guides early EF development, proposing that EF development is rooted in prospective motor control—the ability to plan and guide actions. This theory suggests that EF begins to develop in infancy as the need to control movement through prospective motor control, which is related to the body's preconditions for action. As children age, EF ability differentiates into a separate domain of higher-order action control, with an emphasis on competence in performing motor tasks. Gottwald et al. (2016) support this theory by measuring 18-month-olds' ability to plan actions through the velocity of the first motor unit in reaching within a set of EF tasks, finding a significant link between motor planning and EF task performance. Similar findings linking EF with drawing ability (Riggs et al., 2013; Panesi and Morra, 2016; Simpson et al., 2019) and hand gestures (Rhoads et al., 2018; Zelazo, 2004) in preschoolers further support the role of movement in EF development, especially when considering the developmental trajectory from basic motor actions to more complex cognitive tasks.

Despite promising findings, there is a lack of embodied frameworks for EF development in early childhood (see Thelen and Smith, 1994). This section addresses this gap by examining how existing EF frameworks incorporate embodiment, using Shapiro's (2004, 2007, 2011) three concepts of radical embodied cognition as a guiding structure. These concepts—conceptualization, replacement, and constitution—offer a systematic way to explore how the body, brain, and environment interact in EF development.

## 3 Current frameworks of EF and the role of embodiment

During the preschool years, motor development plays a vital role in cognitive development, particularly in the rapid advancement of EF. EF is broadly understood as a domain-general ability crucial for controlling behavior, thought, and emotion across various contexts (Blair, 2016; Zelazo et al., 1996; Shah and Miyake, 1999). Theoretical frameworks explaining EF development in young children vary in their emphasis on the role of motor skills and bodily preconditions in cognition. These frameworks can be categorized into four main groups: neurocognitive theories, attentional theories, representational theories, and systems theories. This section will:

1. Describe the existing theories of EF,
2. Examine whether these theories address the questions posed by Shapiro's (2011) hypotheses of radical embodied cognition (Table 2), and
3. Evaluate if the current evidence leans more toward perspectives aligned with traditional information-processing or with a more radical embodied cognition approach.

**Table 2:** Description of early EF frameworks and questions of embodied cognition Shapiro, 2011.

	**Neurocognitive**	**Attentional**	**Representational**	**Dynamic systems**
Relationship between body and concepts	The ability to carry out higher-order cognitive processes (i.e., problem-solving, behavioral control) depends primarily on the development of an organism's brain.	The ability to carry out higher-order cognitive processes (i.e., problem-solving, behavioral control) depends largely on the development of an organism's internal attention system.	The ability to carry out higher-order cognitive processes (i.e., problem-solving, behavioral control) primarily depends on the development of an organism's representational ability.	*An organism's body and the surrounding environment directly influence whether concepts can be acquired. Specifically, organisms will utilize multiple systems (movement, internal components, environment) to behave in a particular context (Thelen, 1992; Thelen and Smith, 1994)*.
Need for representations	To execute goal-directed activities, like problem-solving, the prefrontal cortex maintains an internal representation of the goal to send signals for suppressing behaviors that may hinder goal acquisition and coordinate a series of correct behaviors (Funahashi and Andreau, 2013).	Executive functions require the activation of an attentional system for acquiring a rule representation. Errors in executive function may arise when an individual fails to attend to a new rule representation in the environment and instead relies on a previously learned rule.	Executive functions require the formation and active retrieval of task-relevant information which are stored and activated in the prefrontal cortex of the brain. Errors in executive function are a result of a failure to form an abstract representation and instead rely on a stimulus-specific representation (Munakata, 1998; Morton and Munakata, 2002).	*Representations (i.e., symbols that stand for what is being represented that are distinct from the computational forces that operate on them) do not include sensorimotor processes, and therefore, do not fit into a dynamic system. Thus, it is not representation guiding behavior, but rather multiple interacting systems including action, the current environment, and neural connections*.
Mechanisms Involved in Cognition	EF is a set of higher-order internal processes that develop in conjunction with developing regions of the brain, primarily the prefrontal cortex (Luria, 1966; Anderson, 2010).	The development of EF and a central attention system emerge through the input of coupling sensory mechanisms (e.g., looking in the direction that you hear a sound). Therefore, the body serves an assistive role in providing a source of input from the environment.	The key component of executive function is representational ability, of which can be enhanced by external factors like movement, language, and environmental cues. Therefore, the body and environment serve as secondary resources in developing, maintaining, and manipulating mental representations.	*Cognition is the event of several systems binding together in real time. Therefore, temporal coupling of the body, world, and mind is necessary for understanding cognitive processes like executive functioning (Smith, 2005)*.

*Italic font indicates that the component is met;* Upright font indicates that the component is not met.

### 3.1 Neurocognitive theories of EF development

Perhaps the earliest approaches to understanding EF focused on the underlying neurocognitive connections that drive EF development. Such neurocognitive theories of early EF suggest that developmental changes in EF are due to the development of the prefrontal cortex (PFC) of the brain (Diamond, 1988; Diamond and Goldman-Rakic, 1985; Müller and Kerns, 2015; Luria, 1979; Stuss and Benson, 1986; Zelazo and Carlson, 2020; Zelazo, 2020; Perone et al., 2018). The PFC is a region in the anterior portion of the frontal lobe and serves as a key structure for EF (Barbas and Pandya, 1991; Müller and Kerns, 2015; Bjorklund, 1995). This region of the brain is one of the last to reach full maturity with initial rapid development appearing from birth to 2 years of age, and then again between 4 and 7 years, and continued, gradual growth through adolescence followed by a period of neural pruning during early adulthood (Zelazo et al., 2008; Bjorklund, 1995). The role of the brain in the development of EF is integral and cannot be overlooked. As Diamond (2013) and Stuss and Alexander (2000) highlight, neural processes are foundational to executive function. However, EF development does not occur in isolation from bodily actions or environmental contexts. Smith and Gasser (2005) and Wilson (2002) underscore how embodied actions contribute to cognitive processes, while Bronfenbrenner and Morris (2006) and Fisher et al. (2014) demonstrate how environmental interactions shape developmental trajectories. This triadic relationship aligns with Shapiro's (2011) embodied cognition framework, which emphasizes the need to consider brain, body, and environment in a holistic manner when examining EF development.

#### 3.1.1 Body and concept

Neurocognitive theories of EF have traditionally focused on the role of the prefrontal cortex (PFC) in cognitive development (Diamond, 1988; Diamond and Goldman-Rakic, 1985), often neglecting the role of bodily actions. However, to fully understand EF development, it is crucial to examine how bodily actions and sensory experiences specifically shape EF abilities. For instance, Thelen and Smith (1994) highlight that EF, like other cognitive processes, emerges from the intricate interplay between sensory-motor experiences and neural development. They argue that EF development cannot be fully understood without considering how motor actions and sensory interactions with the environment contribute to neural growth and cognitive processes. Thus, the development of EF is deeply interconnected with bodily actions and sensory experiences. The development of the PFC and EF is not isolated from these embodied experiences but is shaped by them. This perspective challenges traditional views that separate brain mechanisms from embodied experiences and supports Shapiro's notion that cognitive functions, including EF, are not solely brain-based but are significantly influenced by interactions between neural processes and bodily actions.

#### 3.1.2 Need for representation

Neurocognitive theories posit a need for internal mediating states (i.e., mental representations) that transform input into behavioral output (Lachman et al., 2015; Shapiro, 2007). While these theories suggest a form of representation in the brain, it is important to clarify that this representation is not necessarily a traditional “mental representation” of a goal. Instead, it may involve distributed elements that concern the body and its interactions with the environment (Wilson, 2002; Chemero, 2011). This form of representation can be considered embodied, as it involves neural processes that are closely linked to bodily experiences and actions (Foglia and Wilson, 2013; Clark, 1999). Therefore, the representation in neurocognitive theories may be more nuanced and distributed than a simple mental representation, and it can be seen as an embodiment of cognitive processes within the brain (Shapiro and Spaulding, 2021).

#### 3.1.3 Cognitive mechanisms

Neurocognitive theories predominantly attribute EF to brain regions, often overlooking the body and environment as significant cognitive factors (Shapiro, 2011). Early neurocognitive models of EF, such as Stuss and Benson's (1986), emphasize the role of the prefrontal cortex (PFC) and surrounding areas in behavioral control and alertness. These models describe how interactions and information processing within these brain regions enable planning, attentional control, monitoring, and response to external stimuli to achieve a goal (Stuss and Benson, 1986; Stuss et al., 1995). Consequently, impairments in executive functioning are often linked to damage in these brain areas (Benton, 1968; Milner, 1963; Stuss and Alexander, 2000).

While early theories emphasize neural activation in the brain as central to EF development, Diamond (1999) highlights the importance of movement and environmental interaction in shaping the neural regions critical for complex cognition. However, much of the literature on EF in early childhood continues to focus primarily on brain mechanisms, with studies like Moriguchi and Hiraki (2013) demonstrating correlations between prefrontal activation and EF abilities in young children. These studies often show abnormal functioning in the prefrontal areas in children with EF-related conditions like attention deficit hyperactivity disorder (ADHD) and autism spectrum disorder (ASD; Moriguchi and Hiraki, 2013).

Although some efforts have been made to integrate movement into neurocognitive theories, these attempts typically serve as extensions to information-processing models. For instance, theories like that of Koziol et al. (2012) and Koziol and Lutz (2013) suggest that the brain evolved to support action, emphasizing interactions between motor-based brain regions (e.g., cerebellum, basal ganglia) and the PFC. While these perspectives acknowledge the importance of the body in cognitive development, they still prioritize internal processing through mental representations as the primary cognitive mechanism. This alignment with information-processing approaches suggests that according to neurocognitive theories, brain processes like those in the PFC primarily drive EF development, regardless of the body's role in environmental interaction and movement.

### 3.2 Attentional theories of development

While neurocognitive theories focus on brain regions, attentional theories of EF development highlight the role of a central attention system in shaping EF abilities (Baddeley, 1986; Norman and Shallice, 1986; Shallice, 1988; Garon et al., 2008; Rothbart et al., 2003). According to Garon et al. (2008), attentional development underpins EF abilities in early childhood. They describe attention as a complex system with two essential subsystems supporting EF development during preschool (Posner and Fan, 2008; Garon et al., 2008, 2014).

The first subsystem, the orienting system, enables shifting attention between stimuli in the environment, allowing for focused attention on specific aspects of an EF task. The second subsystem, the anterior attention system, selects and processes information according to internal representations, aiding in inhibiting irrelevant information and focusing on task-relevant aspects (Ruff and Rothbart, 2001). As these mechanisms develop, children can focus attention longer and process internal and external information more effectively, contributing to EF development (Garon et al., 2008).

#### 3.2.1 Body and concept

Attentional theories of early executive function (EF) development emphasize the role of attention as a mechanism for improving the internal processing of mental representations. These theories suggest that the activation of an attentional system is necessary for acquiring rule representations, which guide behavior (Shallice, 1988). While attentional theories focus on the mental processes of attention and representation, they pay less attention to the relationship between the body and concepts (Shapiro, 2011). Recent research has highlighted the connection between information gathering driven by attention and cognitive skills, such as problem-solving (Ossmy et al., 2020, 2022). This research suggests that attention plays a crucial role in gathering information from the environment to support cognitive processes. By directing attention to relevant stimuli and inhibiting irrelevant information, individuals can enhance their ability to focus on specific aspects of a task (Garon et al., 2008).

#### 3.2.2 Need for representation

Furthermore, attentional theories prioritize attention as a mechanism for improving the internal processing of mental representations (Posner and Petersen, 1990; Rothbart et al., 2003). In attentional theories of EF development, the activation of an attentional system is necessary for acquiring a rule representation (Garon et al., 2008; Diamond, 2013; Zelazo, 2020; see Table 2). Therefore, any errors in executive function may arise when an individual fails to signal a mental representation for attending to appropriate stimuli in the environment and instead relies on a previously learned rule (Perner and Lang, 2002; Munakata et al., 2011.

#### 3.2.3 Cognitive mechanisms

Attentional theories of early executive function (EF) development emphasize an attention network of neural connections that regulate multiple brain regions in service of a goal (Rueda et al., 2004; Garon et al., 2008). While bodily action is not directly accounted for in attentional theories of EF, recent research suggests that embodiment may influence attention and EF development (Wunsch, 2013; Lockman, 2005, 2008; Ossmy et al., 2020).

The development of a core attentional system may rely heavily on motor skill development and engagement with the environment (Friedman et al., 2007; Lempers, 1979). For example, Smith and Gasser (2005) describe the development of attention systems through the coupling of sensory mechanisms, such as when infants look at an event that is producing sound. This interaction between sensory input and motor response highlights that attention is not solely a mental process but requires interactions between the body, mind, and environment to develop (Thelen et al., 2001).

Garon et al. (2014) and Diamond (2013) provide evidence that children's ability to perform more complex EF tasks improves with the development of attentional mechanisms. Many simpler EF tasks are motor-based, requiring the shifting of motor responses based on changes in the environment (Posner and Rothbart, 2007). As tasks become more complex, children utilize attention shifting, which involves shifting between mental representations of the environment. Tasks requiring high motor control may demand lower levels of attentional development, whereas tasks with higher internal processing may rely on the development of both the orienting and the anterior attentional systems (Posner and Rothbart, 2007). While attentional theories do not align with radical embodied perspectives, they may benefit from extending information-processing to incorporate the influence of movement in the development of a central attention system. However, empirical work examining movement within an attentional framework is limited.

### 3.3 Representational theories of EF development

Representational accounts of early EF propose that a primary factor in controlling behavior is representational ability (i.e., mentally describing the environment to link to semantic memory) and reflection (i.e., reprocessing representations to be utilized in working memory). Representational theories propose that internal representations are processed through consciousness in a hierarchical manner (Zelazo, 2004). At the bottom of the hierarchy lies an innate minimal consciousness in that one is aware of the present experience. However, with this level alone, one cannot recall the experience once it has passed. Zelazo (2004) describes minimal consciousness as the process guiding implicit or automatic behaviors as individuals age. The development of the next level of the hierarchy, recursive consciousness, explains the rapid emergence of abilities related to labeling and reflecting on experiences. Specifically, the labeling of an experience allows for the label or information to be stored in working memory and potentially long-term memory. Once the labels are stored, they can be reflected on even when the initial experience has passed. Reflection continues to develop as children gain self-consciousness and abstract rule structure (Zelazo, 2004). Zelazo and colleagues proposed a few reflection based accounts that suggest that reflecting on task relevant representations reduces the likelihood of making an EF error (Marcovitch and Zelazo, 2006, 2009; Zelazo, 2004; Zelazo et al., 2003; Zelazo and Carlson, 2020). For example, Miller and Marcovitch (2011) incorporated labeling cues and visual aids to encourage 2-year-olds' use of representations during an EF task to serve as environmental factors that may enhance reflection on established mental representations of the task. More recently Moriguchi et al. (2015), implemented a paradigm in which preschoolers were randomly assigned to either teach a puppet the rules of an EF task or listen to the rules from an experimenter a second time before completing the critical point of that EF task themselves. The authors hypothesized that children in the conditions that required teaching a puppet the rules of an EF game may have to represent and reflect on the task in different ways than if they were just provided instructions. The results of the study did indicate that children who taught the puppet before playing the game demonstrated an increased performance on the EF task. Perhaps due to the notion that teaching someone requires the development, maintenance, and reflections on mental representations (Moriguchi et al., 2015).

#### 3.3.1 Body and concept

A relationship between the body and concepts as mental representations have started to emerge within representational frameworks (see Table 2). For example, empirical work has examined movement as a method for encouraging the use of representation when cognitive control is needed (e.g., altering reaching patterns to encourage conscious reflection during EF tasks; Marcovitch et al., 2002; Clearfield et al., 2006), which advocates that the body and its interactions with external stimuli influence children's' ability to consciously direct behavior, so much so that movement may need to be included as a component within existing representational frameworks. However, the model does not currently recognize the ability to reflect and maintain mental representations as being body-dependent, which is the premise of conceptualization and the relationship between concepts and the body.

#### 3.3.2 Need for representation

The representational theories of EF development generally oppose radical embodied cognition, primarily because the mechanism underlying EF development within representational theories is mental representation (see Table 2). Specifically, the formation and active retrieval of task-relevant information are stored and activated in the prefrontal cortex of the brain as mental representations. Any errors in executive function are a result of a failure to form an abstract representation and instead rely on a stimulus-specific representation (Munakata, 1998; Morton and Munakata, 2002).

#### 3.3.3 Cognitive mechanisms

Additionally, work has identified that early changes in motor skills may influence children's ability to form representations, therefore directly influencing the development of certain EF ability (Carlson et al., 2005; McClelland and Cameron, 2019), however, the model would identify the constituent of EF as mental representation with movement and contextual factors merely serving as a supplement to internal processing, therefore the cognitive system is composed of internal processes that store and manipulate mental representations (see Table 2).

Although there is evidence supporting components of radical embodied cognition, it is in its early stages and therefore, the role of the body has not been included in the existing representational frameworks. Furthermore, Shapiro (2011) notes that the three themes of radical embodied cognition are not individually exclusive and therefore because replacement is opposed in representational theories of EF development, these theories do not fall under a radical embodied perspective. They do, however, have the potential to incorporate some embodied perspectives within an existing information-processing model, but more work is needed to examine how movement can influence the formation and manipulation of mental representations.

### 3.4 Systems theories of EF development

Systems theories of EF may be the most likely to accommodate radical embodied cognition perspectives. Systems theories of EF development propose that the conscious control of behavior should always be understood in context. More specifically, at the heart of these theories are multiple interacting systems (i.e., genetic, neural, social, motor, etc.) driving behavioral control and developmental change (Perone et al., 2021; Spencer et al., 2012, 2001; Thelen and Smith, 1994; Smith, 2005). A recent systems approach to early EF development proposed by Perone et al. (2021) emphasizes the multi-causal and multi-faceted aspects of goal-directed behavior, stating, for example, that no single factor contributes to proper execution of goal-directed behavior. Thus, systems theories provide a perspective that connects movement to multiple areas of development to describe early EF.

#### 3.4.1 Body and concept

Like Shapiro's radical hypothesis of conceptualization, systems theories suggest that bodily movement within the environment is critical for cognition (see Table 2). Under systems theories, an organism's body and the surrounding environment directly influence whether concepts can be acquired and therefore a multi-component cognitive system is necessary. Specifically, Thelen and Smith (2006) developed a dynamic systems lifespan approach with the foundation being that knowledge is gained from everyday actions. They suggested that behavior is guided by multiple interacting systems that are inherently flexible and self-organize into habit-forming states. Ultimately, proposing that behavior is built around habit (Spencer et al., 2012; Thelen and Smith, 1994). In many EF tasks for example, children receive multiple sources of input (i.e., task demands, context, experimenter cues) to gradually develop a pattern of task behavior. Once there is a change in the task demands, children's behavior is already grounded in a habit, therefore reducing the likelihood of carrying out conscious goal-directed behavior (Thelen et al., 2001).

#### 3.4.2 Need for representation

Furthermore, replacement of the need for mental representations to guide behavior is evident in systems theories (see Table 2). For example, Smith (2005) posits that cognition is embedded within a physical body and world, reducing the need for all knowledge to be stored in the brain through representations. However, it is important to note that the authors of the dynamic systems approach to EF development are strong proponents of mental representation when defined as the mental actions or events that change with changes to the surrounding environment (Thelen et al., 2001).

#### 3.4.3 Cognitive mechanisms

Given the systemic nature of dynamic systems theories, multiple components can be identified as part of the cognitive system (see Table 2). These theories align closely with the hypothesis of constitution in radical embodied cognition, which posits that cognition is the temporal coupling of the body, world, and mental processing (Smith, 2005). Without this real-time interaction among an individual and features of its environment, many cognitive processes cannot be adequately explained.

Dynamic systems theories are particularly robust in conceptualizing the interplay between motor and cognitive systems. Empirical work supporting these theories highlights the bidirectional relationships between motor control and EF. For instance, Gottwald et al. (2016) suggest that EF and motor control develop in the first few years of life with the shared goal of controlling action. Their study, which examined relationships between EF tasks and a prospective motor control task, found significant correlations between simpler EF tasks (e.g., inhibition, working memory) and motor control, supporting the notion that movement is integral to linking higher-order cognition to the current context.

However, dynamic systems theories differ from more traditional EF theories, such as those proposed by Diamond (2013) and Stuss and Alexander (2000), which emphasize the neural and representational mechanisms underlying EF development. These theories prioritize the role of the prefrontal cortex (PFC) and adjacent brain regions in supporting EF but are less explicit in addressing how motor actions and environmental interactions contribute to cognitive development. Embodied cognition perspectives, in contrast, argue for a more integrated view, wherein bodily actions are constitutive of cognition itself rather than merely supportive.

Radical embodied cognition frameworks take this integration further by suggesting that cognitive processes, including EF, cannot be understood in isolation from the agent's active engagement with its environment. While traditional EF theories often conceptualize cognition as occurring “in the head,” dynamic systems and embodied cognition frameworks emphasize the distributed nature of cognition across neural, bodily, and environmental systems.

Despite these advancements, work is still limited in evaluating the integral role movement plays within dynamic systems and radical embodied cognition perspectives of EF development. This gap is particularly evident when considering how these frameworks account for the persistence of EF abilities across the lifespan. Unlike traditional theories, which focus on age-related changes in neural structures, embodied cognition approaches could offer unique insights into how movement-based interventions might support EF at different developmental stages.

Although the theories discussed in this section are separated for the purposes of the proposed review, they share substantial overlap and are not necessarily in conflict. For example, all theories acknowledge the roles of cognitive mechanisms (e.g., attention and representations) and brain regions such as the PFC. However, they differ in the degree to which they incorporate the role of motor control and interaction with the environment. Understanding how an agent's movement within the environment is essential to EF development not only during the critical preschool years but also throughout the lifespan will require further theoretical and empirical exploration.

## 4 Conclusions addressing movement in EF development

Aspects of radical embodied cognition have clearly influenced the four developmental EF theories described in this review, but they vary greatly in the incorporation of the three themes of radical embodied cognition argued to be necessary for truly embodied cognitive research (Shapiro's, 2011). Radical embodied cognition emphasizes the role of bodily actions and environmental interactions in cognitive development, challenging traditional theories that focus predominantly on neural mechanisms (Shapiro's, 2011). Despite its influence, the application of radical embodied cognition to EF development remains relatively nascent (McKenna, 2014).

The integration of radical embodied perspectives with traditional information-processing models reveals both points of convergence and divergence. For instance, dynamic systems theory and traditional information-processing models both recognize the role of cognitive development, but while dynamic systems theory aligns with radical embodied views by emphasizing the interplay of motor actions and cognitive processes (Thelen and Smith, 1994), traditional information-processing models tend to overlook the body's involvement in cognition (Gordon et al., 2021; Lutz and Huitt, 2003). However, one divergence is seen in how working memory is conceptualized: traditional EF theories tend to emphasize internal mental processes, whereas embodied cognition frameworks argue that physical engagement with the environment is fundamental to cognitive control (Shapiro's, 2011). These theoretical differences underscore the need for a more nuanced approach that integrates both embodied and mental representations of EF.

Additionally, while the traditional information-processing models focus heavily on cognitive control mechanisms, they often fail to account for the influence of bodily actions in the development of these skills. For example, embodied cognition frameworks suggest that motor actions not only provide a foundation for cognitive flexibility but also serve as the means by which inhibitory control develops in real-world contexts (Shapiro and Stolz, 2019). This contrast suggests that future research should examine not just the theoretical underpinnings of EF development but also the empirical evidence regarding how these theories apply in different developmental contexts.

Future research should focus on how integrating movement and environmental interactions into EF theories can refine our understanding of cognitive development. This includes exploring how motor activities influence the formation and use of mental representations and assessing the practical implications of these interactions in educational and developmental contexts. Advances in neuroconstructivism and dynamic systems theories could further elucidate the dynamic relationships between the brain, body, and environment (Karmiloff-Smith, 2009). Furthermore, incorporating radical embodied cognition into EF frameworks can provide new insights into how early EF development might be influenced by sensory and motor experiences, which are often overlooked in traditional cognitive models.

While empirical evidence currently does not fully support a radical opposition to traditional information-processing models (Mahon and Caramazza, 2008; Shapiro, 2011), there is growing recognition of the importance of bodily and environmental factors in EF development. This review has highlighted both the strengths and limitations of the current models and has pointed to key areas where these models could benefit from a more integrated approach. Future research should aim to blend insights from both radical embodied cognition and information-processing perspectives, leading to a more comprehensive understanding of cognitive development that acknowledges the interconnectedness of neural, bodily, and environmental factors.

## References

[B1] Anderson P. (2010). Assessment and development of executive function (EF) during childhood. Child Neuropsychol. 8, 71–82. 10.1076/chin.8.2.71.8724 12638061

[B2] Baddeley A. D. (1986). Working Memory. Oxford: Oxford University Press.

[B3] Barbas H. Pandya D. N. (1991). “Patterns of connections of the prefrontal cortex in the rhesus monkey associated with cortical architecture,” in Frontal Lobe Function and Dysfunction, eds. H. S. Levin, H. M. Eisenberg, and A. L. Benton (New York, NY: Oxford University Press), 35–58.

[B4] Benton A. L. (1968). Differential behavioral effects in frontal lobe disease. Neuropsychologia 6, 53–60. 10.1016/0028-3932(68)90038-9

[B5] Bjorklund D. F. (1995). Children's Thinking: Developmental Function and Individual Differences. Pacific Grove, CA: Thomson Brooks/Cole Publishing Co.

[B6] Blair C. (2016). Developmental science and executive function. Curr. Dir. Psychol. Sci. 25, 3–7. 10.1177/0963721415622634 26985139 PMC4789148

[B7] Brandtstädter J. (1998). “Action perspectives on human development,” in Handbook of Child Psychology: Volume 1: Theoretical Models of Human Development, 5th ed, eds. W. Damon, and R. M. Lerner (Hoboken, NJ: Wiley), 807–863.

[B8] Bronfenbrenner U. Morris P. A. (2006). “The bioecological model of human development,” in Handbook of Child Psychology: Vol. 1. Theoretical Models of Human Development, 6th ed, eds. R. M. Lerner and W. Damon (Hoboken, NJ: Wiley), 793–828.

[B9] Carlson S. M. Davis A. C. Leach J. G. (2005). Less is more: executive function and symbolic representation in preschool children. Psychol. Sci. 16, 609–616. 10.1111/j.1467-9280.2005.01583.x 16102063

[B10] Chemero A. (2011). Radical Embodied Cognitive Science. Cambridge, MA: MIT press.

[B11] Chiel H. J. Beer R. D. (1997). The brain has a body: adaptive behavior emerges from interactions of nervous system, body, and environment. Trends Neurosci. 20, 553–557. 10.1016/S0166-2236(97)01149-1 9416664

[B12] Clark A. (1999). An embodied cognitive science? Trends Cogn. Sci. 3, 345–351. 10.1016/S1364-6613(99)01361-3 10461197

[B13] Clark A. (2008). Pressing the flesh: a tension in the study of the embodied, embedded mind? Philos. Phenomenol. Res. 76, 37–59. 10.1111/j.1933-1592.2007.00114.x

[B14] Clearfield M. W. Diedrich F. J. Smith L. B. Thelen E. (2006). Young infants reach correctly in A-not-B tasks: on the development of stability and perseveration. Infant Behav. Dev. 29, 435–444. 10.1016/j.infbeh.2006.03.001 17138296

[B15] Da Rold F. (2018). Defining embodied cognition: the problem of situatedness. New Ideas Psychol. 51, 9–14. 10.1016/j.newideapsych.2018.04.001

[B16] Devine R. T. Ribner A. Hughes C. (2019). Measuring and predicting individual differences in executive functions at 14 months: a longitudinal study. Child Dev. 90:e618–e636. 10.1111/cdev.13217 30663776 PMC6849706

[B17] Diamond A. (1988). Abilities and neural mechanisms underlying A-not-B performance. Child Dev. 59, 523–527. 10.2307/1130330 3359870

[B18] Diamond A. (1999). Development of the ability to use recall to guide action, as indicated by infants' performance on AB. Child Dev. 60, 983–1004. 10.2307/1131038 4042750

[B19] Diamond A. (2013). Executive functions. Annu. Rev. Psychol. 64:135. 10.1146/annurev-psych-113011-143750 23020641 PMC4084861

[B20] Diamond A. Goldman-Rakic P. S. (1985). Evidence for involvement of prefrontal cortex in cognitive changes during the first year of life: comparison of performance of human infant and rhesus monkeys on a detour task with transparent barrier. Soc. Neurosci. Abstr. 11, 832–832.

[B21] Fisher A. V. Godwin K. E. Seltman H. (2014). Visual environment, attention allocation, and learning in young children: when too much of a good thing may be bad. Psychol. Sci. 25, 1362–1370. 10.1177/0956797614533801 24855019

[B22] Foglia L. Wilson R. A. (2013). Embodied cognition. Wiley Interdiscip. Rev. Cogn. Sci. 4, 319–325. 10.1002/wcs.1226 26304209

[B23] Friedman A. H. Watamura S. E. Robertson S. S. (2007). Movement-attention coupling in infancy and attention problems in childhood. Dev. Med. Child Neurol. 47, 660–665. 10.1111/j.1469-8749.2005.tb01050.x 16174308

[B24] Funahashi S. Andreau J. M. (2013). Prefrontal cortex and neural mechanisms of executive function. J. Physiol. 107, 471–482. 10.1016/j.jphysparis.2013.05.001 23684970

[B25] Garon N. Bryson S. E. Smith I. M. (2008). Executive function in preschoolers: a review using an integrative framework. Psychol. Bull. 134, 31–60. 10.1037/0033-2909.134.1.31 18193994

[B26] Garon N. Smith I. M. Bryson S. E. (2014). A novel executive function battery for preschoolers: sensitivity to age differences. Child Neuropsychol. 20, 713–736. 10.1080/09297049.2013.857650 24295496

[B27] Gibson J. J. (1979). “The theory of affordances,” in The People Place and Space Reader, eds. J.J. Gieseking, W. and Mangold (Routledge), 56–60.

[B28] Gordon R. Scalise N. R. Ramani G. B. (2021). Give yourself a hand: the role of gesture and working memory in preschoolers' numerical knowledge. J. Exp. Child Psychol. 208:105145. 10.1016/j.jecp.2021.105145 33848695

[B29] Gottwald J. M. Achermann S. Marciszko C. Lindskog M. Gredebäck G. (2016). An embodied account of early executive-function development: prospective motor control in infancy is related to inhibition and working memory. Psychol. Sci. 27, 1600–1610. 10.1177/0956797616667447 27765900 PMC5154392

[B30] Hoch J. E. Adolph K. E. (2019). Motor development: embodied, embedded, enculturated, and enabling. Annu. Rev. Psychol. 70, 129–153. 10.1146/annurev-psych-010418-102836 30256718 PMC6320716

[B31] Hohmann M. Weikart D. P. Epstein A. S. (1995). Educating Young Children: Active Learning Practices for Preschool and Child Care Programs. Ypsilanti, MI: High/Scope Press.

[B32] Hutchins E. (1995). Cognition in the Wild. Cambridge, MA: The MIT Press. 10.7551/mitpress/1881.001.0001

[B33] Karmiloff-Smith A. (2009). Neurocognitive Development: Developmental Trajectories and Their Implications. New York, NY: Psychology Press.

[B34] Kirsh D. (1995). The intelligent use of space. Artif. Intell. 73, 31–68. 10.1016/0004-3702(94)00017-U

[B35] Kirsh D. (2010). Thinking with external representations. AI Society 25, 441–454. 10.1007/s00146-010-0272-8

[B36] Koziol L. F. Budding D. E. Chidekel D. (2012). From movement to thought: executive function, embodied cognition, and the cerebellum. Cerebellum 11, 505–525. 10.1007/s12311-011-0321-y 22068584

[B37] Koziol L. F. Lutz J. T. (2013). From movement to thought: the development of executive function. Appl. Neuropsychol. Child 2, 104–115. 10.1080/21622965.2013.748386 23848244

[B38] Lachman R. Lachman J. L. Butterfield E. C. (2015). Cognitive Psychology and Information Processing: An Introduction. New York, NY: Psychology Press.

[B39] Lally M. Valentine-French S. (2019). Lifespan Development: A Psychological Perspective, 2nd Edn. Grayslake, IL: College of Lake County.

[B40] Lempers J. D. (1979). Young children's production and comprehension of nonverbal deictic behaviors. J. Genet. Psychol. 135, 93–102. 10.1080/00221325.1979.10533420 556593

[B41] Lerner R. M. Agans J. P. DeSouza L. M. Hershberg R. M. (2017). The end of the beginning: evidence and absences studying positive youth development in a global context. Adolesc. Res. Rev. 2, 1–14. 10.1007/s40894-016-0046-z

[B42] Lerner R. M. Schmid Callina K. (2013). Relational developmental systems theories and the ecological validity of experimental designs. Hum. Dev. 56, 372–380. 10.1159/000357179

[B43] Lockman J. J. (2000). A perception-action perspective on tool use development. Child Dev. 71, 137–144. 10.1111/1467-8624.00127 10836567

[B44] Lockman J. J. (2005). “Tool use as an emergent phenomenon,” in Action as an Organizer of Learning and Development, eds. J. J. Rieser, J. J. Lockman, and C. A. Nelson (Mahwah, NJ: Lawrence Erlbaum Associates), 131–150.

[B45] Lockman J. J. (2008). The development of manual skills. Annu. Rev. Psychol. 59, 613–634. 10.1146/annurev.psych.59.103006.093618

[B46] Luria A. R. (1966). Higher Cortical Functions in Man. New York, NY: Basic Books.

[B47] Luria A. R. (1979). The Making of Mind: A Personal Account of Soviet Psychology. Cambridge, MA: Harvard University Press.

[B48] Lutz S. Huitt W. (2003). Information processing and memory: theory and applications. Educ. Psychol. Interact. 1−17. Available at: http://www.edpsycinteractive.org/papers/infoproc.pdf

[B49] Lux V. Marshall P. J. Shing Y. L. (2021). The role of sleep in the development of memory consolidation. Dev. Rev. 60:100961. 10.1016/j.dr.2021.100961

[B50] Mahon B. Z. Caramazza A. (2008). A critical look at the embodied cognition hypothesis and a new proposal for grounding conceptual content. J. Physiol. 102, 59–70. 10.1016/j.jphysparis.2008.03.004 18448316

[B51] Marcovitch S. Zelazo P. D. (2006). The influence of number of A trials on 2-years-old's behavior in two A-Not-B-type search tasks: a test of the hierarchical competing systems model. J. Cogn. Dev. 7, 477–501. 10.1207/s15327647jcd0704_3

[B52] Marcovitch S. Zelazo P. D. (2009). A hierarchical competing systems model of the emergence and early development of executive function. Dev. Sci. 12, 1–25. 10.1111/j.1467-7687.2008.00754.x 19120405 PMC2842568

[B53] Marcovitch S. Zelazo P. D. Schmuckler M. A. (2002). The effect of the number of A trials on performance on the A-not-B task. Infancy 3, 519–529. 10.1207/S15327078IN0304_06

[B54] Marshall P. J. (2016). Embodiment and human development. Child Dev. Perspect. 10, 245–250. 10.1111/cdep.12190 27833651 PMC5098554

[B55] Martiny K. M. (2011). Book review of Lawrence Shapiro's embodied cognition. Phenomenol. Cogn. Sci. 10, 297–305. 10.1007/s11097-011-9199-x

[B56] McClelland M. M. Cameron C. E. (2019). Developing together: the role of executive function and motor skills in children's early academic lives. Early Child. Res. Q. 46, 142–151. 10.1016/j.ecresq.2018.03.014

[B57] McKenna P. (2014). Are executive functions embodied? A brief theoretical and empirical discussion. PsyPAG Q. 37–41. 10.53841/bpspag.2014.1.91.34

[B58] Miller S. E. Marcovitch S. (2011). Toddlers benefit from labeling on an executive function search task. J. Exp. Child Psychol. 108, 580–592. 10.1016/j.jecp.2010.10.008 21112597 PMC3042530

[B59] Milner B. (1963). Effect of different brain lesions on card sorting. JAMA Neurology 9, 90–100. 10.1001/archneur.1963.00460070100010

[B60] Miyake A. Friedman N. P. Emerson M. J. Witzki A. H. Howerter A. Wager T. D. (2000). The unity and diversity of executive functions and their contributions to complex “frontal lobe” tasks: a latent variable analysis. Cogn. Psychol. 41, 49–100. 10.1006/cogp.1999.0734 10945922

[B61] Moriguchi Y. Hiraki K. (2013). Prefrontal cortex and executive function in young children: a review of NIRS studies. Front. Hum. Neurosci. 7:867. 10.3389/fnhum.2013.00867 24381551 PMC3865781

[B62] Moriguchi Y. Sakata Y. Ishibashi M. Ishikawa Y. (2015). Teaching others rule-use improves executive function and prefrontal activations in young children. Front. Psychol. 6:894. 10.3389/fpsyg.2015.00894 26175706 PMC4484979

[B63] Morton J. B. Munakata Y. (2002). Active versus latent representations: a neural network model of perseveration, dissociation, and decalage. Dev. Psychobiol. 40, 255–265. 10.1002/dev.10033 11891637

[B64] Müller U. Kerns K. (2015). “The development of executive function,” in Handbook of Child Psychology and Developmental Science: Cognitive Processes, eds. L. S. Liben, U. Müller, and R. M. Lerner (John Wiley and Sons, Inc.), 571–623. 10.1002/9781118963418.childpsy214

[B65] Munakata Y. (1998). Infant perseveration and implications for object permanence theories: a PDP model of the A-not-B task. Dev. Sci. 1, 161–184. 10.1111/1467-7687.00021

[B66] Munakata Y. Snyder H. R. Chatham C. H. (2011). Developing cognitive control: three key transitions. Curr. Dir. Psychol. Sci. 21, 71–77. 10.1177/0963721412436807 22711982 PMC3375849

[B67] Norman D. A. Shallice T. (1986). “Attention to action,” in Consciousness and Self-regulation (Springer, Boston, MA), 1–18. 10.1007/978-1-4757-0629-1_1

[B68] Ossmy O. Ben-Shachar M. Mukamel R. (2020). The neural basis of reading acquisition: a cross-sectional study of Hebrew children. Neuroimage 207:116350. 10.1016/j.neuroimage.2019.116350 31733373

[B69] Ossmy O. Ben-Shachar M. Mukamel R. (2022). Neural correlates of reading acquisition in Hebrew: a longitudinal study. Neuroimage 247:118830. 10.1016/j.neuroimage.2021.118830 34965454 PMC9379864

[B70] Panesi S. Morra S. (2016). Drawing a dog: the role of working memory and executive function. J. Exp. Child Psychol. 152, 1–11. 10.1016/j.jecp.2016.06.015 27454235

[B71] Pellegrini A. D. Smith P. K. (1998). Physical activity play: the nature and function of a neglected aspect of play. Child Dev. 69, 577–598. 10.1111/j.1467-8624.1998.tb06226.x 9680672

[B72] Pellegrino J. W. Goldman S. R. (1987). Information processing and elementary mathematics. J. Learn. Disabil. 20, 23–32. 10.1177/002221948702000106 3805888

[B73] Perner J. Lang B. (2002). What causes 3-year-olds' difficulty on the dimensional change card sorting task? Infant Child Dev. 11, 93–105. 10.1002/icd.299

[B74] Perone S. Almy B. Zelazo P. D. (2018). “Toward an understanding of the neural basis of executive function development,” in The Neurobiology of Brain and Behavioral Development, eds. K. J. S. Anand and M. K. L. N. R. Dubey (San Diego, CA: Academic Press), 291–314. 10.1016/B978-0-12-804036-2.00011-X

[B75] Perone S. Simmering V. R. Buss A. T. (2021). A dynamical reconceptualization of executive-function development. Perspect. Psychol. Sci. 16, 1198–1208. 10.1177/1745691620966792 33593126 PMC8364921

[B76] Piaget J. (1936). Origins of Intelligence in the Child. London: Routledge and Kegan Paul.

[B77] Piaget J. (1959). Language and Thought of the Child. New York: Humanities Press.

[B78] Piaget J. (1971). “*The theory of stages in cognitive development*,” in Measurement and Piaget, eds. D. R. Green, M. P. Ford, and G. B. Flamer (New York, NY: McGraw-Hill).

[B79] Posner M. I. Fan J. (2008). “Attention as an organ system,” in Topics in Integrative Neuroscience: From Cells to Cognition, ed. J. Pomerantz (Cambridge University Press), 31–61. 10.1017/CBO9780511541681.005

[B80] Posner M. I. Petersen S. E. (1990). The attention system of the human brain. Annu. Rev. Neurosci. 13, 25–42. 10.1146/annurev.ne.13.030190.000325 2183676

[B81] Posner M. I. Rothbart M. K. (2007). Attention in Development: From Theory to Practice. New York, NY: Oxford University Press.

[B82] Rhoads C. L. Miller P. H. Jaeger G. O. (2018). Put your hands up! *Gesturing improves preschoolers' executive function. J. Exp. Child Psychol*. 173, 41–58. 10.1016/j.jecp.2018.03.010 29677552

[B83] Ridler K. Veijola J. M. Tanskanen P. Miettunen J. Chitnis X. Suckling J.. (2006). Fronto-cerebellar systems are associated with infant motor and adult executive functions in healthy adults but not in schizophrenia. Proc. Natl. Acad. Sci. 103, 15651–15656. 10.1073/pnas.0602639103 17028177 PMC1636802

[B84] Riggs K. J. Jolley R. P. Simpson A. (2013). The role of inhibitory control in the development of human figure drawing in young children. J. Exp. Child Psychol. 114, 537–542. 10.1016/j.jecp.2012.10.003 23313449

[B85] Rothbart M. K. Ellis L. K. Rosario Rueda M. Posner M. I. (2003). Developing mechanisms of temperamental effortful control. J. Pers. 71, 1113–1144. 10.1111/1467-6494.7106009 14633060

[B86] Rueda M. R. Posner M. I. Rothbart M. K. (2004). “Attentional control and self-regulation,” in Handbook of Self-regulation: Research, Theory, and Applications, eds. R. F. Baumeister and K. D. Vohs (New York, NY: The Guilford Press), 283–300.

[B87] Ruff H. A. Rothbart M. K. (2001). Attention in Early Development: Themes and Variations. New York, NY: Oxford University Press.

[B88] Shah P. Miyake A. (1999). “Models of working memory: an introduction,” in Models of Working Memory: Mechanisms of Active Maintenance and Executive Control, eds. A. Miyake and P. Shah (Cambridge: Cambridge University Press), 1–27. 10.1017/CBO9781139174909.004

[B89] Shallice T. (1988). From Neuropsychology to Mental Structure. Cambridge, UK: Cambridge University Press. 10.1017/CBO9780511526817

[B90] Shapiro K. (2019). The Cambridge Handbook of Working Memory and Language. Cambridge: Cambridge University Press.

[B91] Shapiro L. (2004). The Mind Incarnate. Cambridge, MA: The Massachusetts Institute of Technology (MIT) Press.

[B92] Shapiro L. (2007). The embodied cognition research programme. **Philos. Compass**. 2, 338–346. 10.1111/j.1747-9991.2007.00064.x

[B93] Shapiro L. (2011), Embodied Cognition. London and New York: Routledge.

[B94] Shapiro L. (2012). “Embodied cognition,” in The Oxford Handbook of Philosophy of Cognitive Science, eds. E. Margolis, R. Samuels, and S. Stich (Oxford: Oxford University Press), 118–146.

[B95] Shapiro L. Spaulding S. (2021). The Routledge Handbook of Embodied Cognition. New York, NY: Routledge.

[B96] Shapiro L. Stolz S. A. (2019). Embodied cognition and its significance for education. Theory Res. Educ. 17, 19–39. 10.1177/1477878518822149

[B97] Simpson A. Al Ruwaili R. Jolley R. Leonard H. Geeraert N. Riggs K. J. (2019). Fine motor control underlies the association between response inhibition and drawing skill in early development. Child Dev. 90, 911–923. 10.1111/cdev.12949 28902393

[B98] Smith L. Gasser M. (2005). The development of embodied cognition: six lessons from babies. Artif. Life 11, 13–29. 10.1162/1064546053278973 15811218

[B99] Smith L. B. (2005). Cognition as a dynamic system: principles from embodiment. Dev. Rev. 25, 278–298. 10.1016/j.dr.2005.11.001

[B100] Spencer J. P. Austin A. Schutte A. R. (2012). Contributions of dynamic systems theory to cognitive development. Cogn. Dev. 27, 401–418. 10.1016/j.cogdev.2012.07.006 26052181 PMC4454421

[B101] Spencer J. P. Smith L. B. Thelen E. (2001). Tests of a dynamic systems account of the A not B error: the influence of prior experience on the spatial memory abilities of two year olds. Child Dev. 72, 1327–1346. 10.1111/1467-8624.00351 11699674

[B102] Stuss D. T. Alexander M. P. (2000). Executive functions and the frontal lobes: a conceptual view. Psychol. Res. 63, 289–298. 10.1007/s004269900007 11004882

[B103] Stuss D. T. Benson D. F. (1984). Neuropsychological studies of the frontal lobes. Psychol. Bull. 95:3. 10.1037/0033-2909.95.1.3 6544432

[B104] Stuss D. T. Benson D. F. (1986). The Frontal Lobes. New York, NY: Raven Press.

[B105] Stuss D. T. Shallice T. Alexander M. P. Picton T. W. (1995). A multidisciplinary approach to anterior attentional *functions Ann. NY Acad. Sci*. 769:191–211. 10.1111/j.1749-6632.1995.tb38140.x 8595026

[B106] Thelen E. (1992). Motor Development: A New Synthesis. Cambridge, MA: MIT Press.

[B107] Thelen E. Schöner G. Scheier C. Smith L. B. (2001). The dynamics of embodiment: a field theory of infant perseverative reaching. Behav. Brain Sci. 24, 1–34. 10.1017/S0140525X01003910 11515285

[B108] Thelen E. Smith L. B. (1994). “A dynamic systems approach to the development of cognition and action,” in MIT Press/Bradford Book Series in Cognitive Psychology. Cambridge, MA, US: The MIT Press. 10.7551/mitpress/2524.001.0001

[B109] Thelen E. Smith L. B. (2006). “Dynamic systems theories,” in Handbook of Child Psychology, 6th Edn, Vol. 1, eds. R. M. Lerner and W. Damon (Wiley), 258–312.

[B110] Varela F. J. Thompson E. Rosch E. (2016). The Embodied Mind: Cognitive Science and Human Experience. Cambridge, MA: MIT Press. 10.7551/mitpress/9780262529365.001.0001

[B111] Vygotsky L. S. (1962). Thought and language, eds. E. Hanfmann and G. Vakar, and trans. (Cambridge, MA: MIT Press).

[B112] Vygotsky L. S. (1978). Mind in Society: The Development of Higher Mental Psychological Processes, eds. M. Cole, V. John-Steiner, S. Scribner, and E. Souberman, (Cambridge, MA: Harvard University Press).

[B113] Wilson A. D. Golonka S. (2013). Embodied cognition is not what you think it is. Front. Psychol. 4:58. 10.3389/fpsyg.2013.00058 23408669 PMC3569617

[B114] Wilson M. (2002). Six views of embodied cognition. Psychon. Bull. Rev. 9, 625–636. 10.3758/BF03196322 12613670

[B115] Wilson R. A. (1994). Wide computationalism. Mind 103, 351–372. 10.1093/mind/103.411.351

[B116] Wunsch K. (2013). The role of executive functions in the development of motor skills in children. Eur. J. Dev. Psychol. 10, 526–544. 10.1080/17405629.2013.797338

[B117] Zelazo P. D. (2004). The development of conscious control in childhood. Trends Cogn. Sci. 8, 12–17. 10.1016/j.tics.2003.11.001 14697398

[B118] Zelazo P. D. (2020). Executive function and psychopathology: a neurodevelopmental perspective. Annu. Rev. Clin. Psychol. 16, 431–454. 10.1146/annurev-clinpsy-072319-024242 32075434

[B119] Zelazo P. D. Carlson S. M. (2012). Hot and cool executive function in childhood and adolescence: development and plasticity. Child Dev. Perspect. 6, 354–360. 10.1111/j.1750-8606.2012.00246.x

[B120] Zelazo P. D. Carlson S. M. (2020). The neurodevelopment of executive function skills: implications for academic achievement gaps. Psychol. Neurosci. 13, 273–298. 10.1037/pne0000208

[B121] Zelazo P. D. Carlson S. M. Kesek A. (2008). “The development of executive function in childhood,” in Handbook of Developmental Cognitive Neuroscience, Eds. C. A. Nelson and M. Luciana (Boston Review), 553–574.

[B122] Zelazo P. D. Frye D. Rapus T. (1996). An age-related dissociation between knowing rules and using them. Cogn. Dev. 11, 37–63. 10.1016/S0885-2014(96)90027-1

[B123] Zelazo P. D. Müller U. Frye D. Marcovitch S. Argitis G. Boseovski J.. (2003). The development of executive function in early childhood. Monogr. Soc. Res. Child Dev. 68, i-151. 10.1111/j.0037-976X.2003.00261.x 14723273

